# Delivering information: A descriptive study of Australian women’s information needs for decision-making about birth facility

**DOI:** 10.1186/1471-2393-12-51

**Published:** 2012-06-18

**Authors:** Rachel Thompson, Aleena M Wojcieszek

**Affiliations:** 1 Queensland Centre for Mothers and Babies, School of Psychology, The University of Queensland, Brisbane, Queensland, 4072, Australia

**Keywords:** Maternity care, Information needs, Decision-making, Informed choice

## Abstract

**Background:**

Little information is known about what information women want when choosing a birth facility. The objective of this study was to inform the development of a consumer decision support tool about birth facility by identifying the information needs of maternity care consumers in Queensland, Australia.

**Methods:**

Participants were 146 women residing in both urban and rural areas of Queensland, Australia who were pregnant and/or had recently given birth. A cross-sectional survey was administered in which participants were asked to rate the importance of 42 information items to their decision-making about birth facility. Participants could also provide up to ten additional information items of interest in an open-ended question.

**Results:**

On average, participants rated 30 of the 42 information items as important to decision-making about birth facility. While the majority of information items were valued by most participants, those related to policies about support people, other women’s recommendations about the facility, freedom to choose one’s preferred position during labour and birth, the aesthetic quality of the facility, and access to on-site neonatal intensive care were particularly widely valued. Additional items of interest frequently focused on postnatal care and support, policies related to medical intervention, and access to water immersion.

**Conclusions:**

The women surveyed had significant and diverse information needs for decision-making about birth facility. These findings have immediate applications for the development of decision support tools about birth facility, and highlight the need for tools which provide a large volume of information in an accessible and user-friendly format. These findings may also be used to guide communication and information-sharing by care providers involved in counselling pregnant women and families about their options for birth facility or providing referrals to birth facilities.

## Background

In Australia, the overwhelming majority of women give birth in conventional hospital or birth centre settings (96.9 % and 2.2 %, respectively), with less than 1 % of women giving birth at home or in other settings [1]. There is significant variation in the nature of the maternity care provided by different hospitals and birth centres in Australia. For example, there is marked variation in the clinical capabilities of different facilities. Facilities range from those intended to provide care only for healthy women with pregnancies regarded as ‘low risk’, which may not be equipped to provide on-site access to medical procedures such as epidural anaesthesia or caesarean section (e.g., co-located or free-standing birth centres, small rural hospitals), through to those equipped to provide care to all women, including those with ‘high risk’ pregnancies and complex care needs (i.e., tertiary referral centres) [2].

There is also variation in the models of maternity care offered by Australian birth facilities. Within public sector facilities, several models of care including general practitioner (GP) shared care, team midwifery care, caseload (or group practice) midwifery care, conventional public care, and birth centre care may be available at a birth facility. Alternatively, private sector facilities typically offer only a single model where care is managed by a private specialist obstetrician [3-5].

Birth facilities also vary widely in their policies and practices, including in their rates of clinical procedures, even when risk-adjusted data are considered (that is, when the influence of patient mix is minimised). For example, in one Australian state in 2008, the rate of induction of labour for standard primiparae^a^ varied from 0 % to 23 % within public facilities, and also varied considerably between public facilities and private facilities overall (4 % and 13 %, respectively) [6]. The caesarean section rate for the same sample of women also varied from 0 % to 31 % within public facilities, and between public facilities and private facilities overall (16 % and 25 %, respectively) [6].

Clinical outcomes for mothers and babies also vary across birth facilities and, again, even when risk-adjusted data are considered. For example, the rate of third- and fourth-degree perineal tears sustained by standard primiparae giving birth vaginally varied from 0 % to 17 % within public facilities in one state of Australia in 2008, and also varied considerably between private facilities and public facilities overall (2 % and 5 %, respectively) [6]. Special care nursery (SCN) or neonatal intensive care unit (NICU) admission rates for term infants without birth defects also varied from 0 % to 53 % within public facilities [6].

Inter-facility differences in clinical capabilities, models of care, policies and practices, and maternal and infant outcomes are accompanied by inter-facility differences in consumer ratings of the quality of maternity care. For example, data from a population survey of recent maternity care consumers in Queensland, Australia demonstrated that ratings of the quality of interpersonal care varied widely across public facilities; the proportion of women reporting being cared for ‘very well’ during labour and birth ranged from 53 % to 100 % [7]. Several other inter-facility variations in women’s self-reported experiences were also observed, including in the proportion of women that said that they were always treated with kindness and understanding (ranging from 68 % to 100 % within public facilities), the proportion that said that their decisions were always respected (ranging from 56 % to 100 % within public facilities), and the proportion that said they would recommend the birth facility to a friend (ranging from 77 % to 100 % within public facilities) [7].

The existence of numerous birth facility options, together with significant variability between facilities in attributes and clinical capabilities, highlights the importance of supporting maternity care consumers to make informed decisions about where to birth. In particular, it suggests the potential value of decision support tools which could provide consumers with information about different facilities and the care they offer, and enable them to identify the birth facility that best meets their individual needs, preferences and values. Such decision support tools would not only respond to the ethical imperative to support consumers to make informed decisions about where to birth, but would also respond to consumer dissatisfaction with the limited availability of reliable information on maternity care choices [4,8]. In circumstances where birth facility options may be more limited, the content of decision support tools may still allow women to develop more accurate expectations, and feel better prepared, for their intrapartum care.

Decision support tools about birth facility have potential to respond to an important need, and evaluations of such tools in different settings have highlighted their effectiveness for facilitating effective decision-making [9]. However, the utility of decision support tools depends on the extent to which they provide users with relevant and valued information [10]. Understanding consumers’ information needs for decision-making about birth facility is, therefore, critical to the development of useful decision support tools. However, we currently lack comprehensive knowledge of women’s information needs for decision-making about birth facility, as we discuss below.

Although research has been conducted in several countries to determine the attributes that consumers prefer in a maternity care facility e.g., [11,12] and to determine the criteria that maternity care consumers use in selecting a maternity hospital e.g., [13], these studies have typically measured consumer preferences on only a limited number of attributes. Hundley et al., for example, examined the preferences of pregnant women in Scotland on six key attributes of intrapartum care (i.e., type of midwifery care, options for pain relief, practices in fetal monitoring, ‘homeliness’ of the birth environment, medical staff involvement, and approach to decision-making) [11]. Combier et al. surveyed postnatal women in France to determine which out of list of nine criteria (i.e., ease of access, proximity, medical advice or recommendation, satisfaction in previous care, recommendations from family/friends, comfort/physical conditions, quality of contact with personnel, technical quality, and cost) they had used when previously selecting a maternity facility [13]. The importance of a range of other attributes, however, remains untested. Moreover, the findings of much previous research have lacked sufficient specificity to be useful in informing the content of decision support tools.

Other studies e.g., [14-17] have examined predictors of consumer satisfaction with maternity care to shed light on the valued attributes of care. These studies are important both for maternity service planning and for the prioritisation of quality improvement efforts. However, their findings have only limited relevance to the development of decision support tools for prospective decision-making about birth facility, as attributes that predict high retrospective consumer satisfaction with maternity services may differ from those that are prioritised by women when choosing between birth facilities.

Given our intention to develop a consumer decision support tool on birth facility for women residing in Queensland, Australia, and the limited evidence to inform the content of such a tool, we sought to examine women’s information needs when deciding between birth facilities. This descriptive paper summarises the findings of a cross-sectional survey in which women in Queensland who were pregnant and/or had recently had a baby were asked to rate the relative importance of 42 different information items to decision-making about birth facility. Participants’ open-ended responses when invited to specify further items of importance to decision-making about birth facility were also elicited to ensure study findings were not constrained by the information items listed in the survey, and these are also described.

## Methods

### Participants

Participants comprised a convenience sample of 146 women residing in both urban and rural regions in Queensland, Australia, who were pregnant and/or had recently had a baby (i.e., in the last two years). Several strategies were implemented to recruit women to participate in the study. We placed posters advertising the study and providing the address of the study website (which contained an information sheet, online background questionnaire and online survey) in childcare centres, shopping centres, postnatal clinics, and other community settings in urban areas of Queensland. Posters advertising the study and providing the address of the study website were also placed in several public antenatal clinics, birth facilities, and new parent groups in rural areas of Queensland. In these settings, some women were also recruited face-to-face by research assistants and could complete a hard copy of the background questionnaire and survey either immediately or via reply-paid return mail. We also distributed electronic study invitations providing the study website address to an email list of maternity care consumers residing across Queensland and via maternity care stakeholder networks.

### Procedure

Ethical approval for the study was obtained from The University of Queensland Behavioural & Social Sciences Ethical Review Committee (BSSERC). Completion of the survey either in hard copy or online was taken as informed consent to participate in the study. Data were collected from April to June, 2010.

### Measures

#### *Background questionnaire*

A questionnaire was administered simultaneously with the survey of information needs to collect data on participants’ background characteristics including age, pregnancy status, number and ages of children, country of birth, language spoken at home, indigenous identification and highest level of education. Responses pertaining to country of birth and language spoken at home were coded using the Standard Australian Classification of Countries [18] and the Australian Standard Classification of Languages [19], respectively.

#### *Survey of information needs*

A survey was developed in which participants were presented with a prepared list of information items that represented questions potentially relevant to decision-making about birth facility. Items were derived from reviews of relevant published literature e.g., [11-13], the findings of consultation with current and recent maternity care consumers (e.g., face to face consultation at community events, consultation with a consumer advisory group), and an examination of existing maternity care decision support tools (e.g., the NHS Pregnancy Care Planner [20]). In developing the survey, we endeavoured to select items important to a broad range of maternity care consumers. Pilot testing of the survey was deemed unnecessary due to its straightforward nature, and the survey was designed to allow participants to highlight any items they did not understand.

Participants were asked to rate the importance of each information item to their decision-making about where to birth, using a five-point scale (1 = extremely unimportant, 2 = unimportant, 3 = neutral, 4 = important, 5 = extremely important). In the event that they were unsure of the meaning of a particular item, participants could specify “I don’t know what this means”. In order to minimise participant burden and to maximise data quality, the list of items provided to participants was limited to 42. However, participants were also provided with an open-ended section in which they could specify up to ten further items of importance to decision-making about birth facility.

### Analytic strategy

Descriptive statistical analyses were conducted to calculate the percentage of participants who rated each information item as ‘important’ (4) or ‘extremely important’ (5) to decision-making about birth facility. Analyses were conducted using the Statistical Package for the Social Sciences (SPSS) Version 20. Participants’ open-ended responses when invited to specify further items of key personal importance to decision-making about birth facility were coded into categories by both authors independently. Any disagreements were resolved by discussion. Once categories had been identified, the number of participants providing at least one response in each category was calculated, so that multiple similar responses by an individual participant would not overinflate the importance to the sample of a particular category.

## Results

### Participant characteristics

A total of 174 women completed the survey. After excluding women who were not pregnant and who did not have a baby aged 2 years or younger (n = 10), and women who did not provide data to allow their eligibility to be confirmed (n = 18), the final eligible sample comprised 146 participants. Most participants were aged 30–34 years (41.1 %), 25–29 years (24.0 %) or 35–39 years (21.9 %; see Table 1). Approximately 38 % of participants were pregnant at the time of completing the study and participants most commonly reported having had one previous birth (45 %). The majority of participants was born in Australia (82.2 %), spoke English at home (94.5 %), identified as neither Aboriginal nor Torres Strait Islander (97.3 %), and had a Bachelor or other degree (73 %). Due to the recruitment strategy adopted in this study, neither the number of women exposed to the invitation to participate nor the survey response rate could be determined.

**Table 1:** Participant background characteristics (n=146)

	**Freq**	**(%)**
Age (years)		
<20 years	3	(2.1%)
20-24 years	5	(3.4%)
25-29 years	35	(24.0%)
30-34 years	60	(41.1%)
35-39 years	32	(21.9%)
>39 years	11	(7.5%)
Pregnancy status		
Pregnant	56	(38.4%)
Not pregnant	90	(61.6%)
Had baby in the last two years		
Yes	107	(73.8%)
No	38	(26.2%)
* Missing*	1	
Parity		
0	30	(20.7%)
1	65	(44.8%)
2	34	(23.4%)
3+	16	(11.0%)
* Missing*	1	
Country of birth		
Australia	120	(82.2%)
Other Oceania and Antarctica	6	(4.1%)
North-West, Southern and Eastern Europe	9	(6.2%)
Americas	4	(2.7%)
Sub-Saharan Africa	3	(2.1%)
South-East, Southern and Central Asia	4	(2.8%)
Language spoken at home		
English	138	(94.5%)
Other Northern European Languages	3	(2.1%)
Southern European Languages	3	(2.1%)
Eastern European Languages	1	(0.7%)
Eastern Asian Languages	1	(0.7%)
Indigenous identification		
Aboriginal only	4	(2.7%)
Torres Strait Islander only	0	(0%)
Aboriginal and Torres Strait Islander	0	(0%)
Neither Aboriginal nor Torres Strait Islander	142	(97.3%)
Highest level of education		
Less than Year 10 or equivalent	1	(0.7%)
Year 10 or equivalent	1	(0.7%)
Year 11/12 or equivalent	15	(10.3%)
Trade certificate or other certificate	7	(4.8%)
Associate diploma or other diploma	15	(10.3%)
Bachelor or other degree	107	(73.3%)

### Ratings of the importance of information items

On average, participants rated 30.1 items (SD = 0.5; range = 16–41) as either important or extremely important to decision-making about birth facility. While the majority of information items were important to most participants, some information items were particularly widely valued (see Table 2).

**Table 2:** Proportion of participants who rated each information item as ‘important’ or ‘extremely important’ (n=146)

	**Important**		**Extremely Important**		**Important or Extremely Important**	
**Information Item**	**Freq**	**%**	**Freq**	**%**	**Freq**	**%**
12. Can my support people (e.g., partner, companion) be in the birth room when I’m having my baby?	5	3.4%	141	96.6%	146	100.0%
10. Would other women recommend this facility to their friends?	57	39.0%	84	57.5%	141	96.6%
17. Can I choose what position I want to be in during my labour and birth here (e.g., squatting, sitting, moving around)?	28	19.2%	112	76.7%	140	95.9%
7. How ‘nice’ is this facility (e.g., clean, 'new', etc)?	69	47.3%	67	45.9%	136	93.2%
38. Will my support people (e.g., partner, companion) be made to feel welcome?	61	41.8%	74	50.7%	135	92.5%
31. Does this facility have an intensive care unit for babies (i.e., a ‘NICU’)?	32	22.1%	101	69.7%	133	91.7%
20. What models of care are offered at this facility?	62	44.9%	62	44.9%	124	89.9%
2. Is this facility close to my home?	66	45.2%	64	43.8%	130	89.0%
41. Can I discuss my birth plan with my care provider?	61	42.4%	66	45.8%	127	88.2%
13. Can my partner stay overnight (and is there a cost for this)?	34	23.3%	94	64.4%	128	87.7%
28. What methods of pain relief can I have?	56	38.4%	72	49.3%	128	87.7%
6. How many care providers are there per woman at this facility?	67	45.9%	59	40.4%	126	86.3%
25. Are classes about how to breastfeed my baby offered?	57	39.3%	67	46.2%	124	85.5%
9. How many nights after birth can I stay?	74	51.0%	49	33.8%	123	84.8%
42. How many women have skin-to-skin contact with their baby straight after birth?	28	19.3%	94	64.8%	122	84.1%
4. Are tours of the facility available beforehand?	76	52.1%	43	29.5%	119	81.5%
18. Can I ask to have an epidural for pain management?	44	30.1%	74	50.7%	118	80.8%
24. Are prenatal and postnatal classes offered here?	73	50.0%	44	30.1%	117	80.1%
27. Can I have a private room?	47	32.2%	69	47.3%	116	79.5%
30. Can this facility provide “high-tech” care?	34	25.8%	70	53.0%	104	78.8%
19. Can I have a vaginal birth at this facility if I have had a caesarean section ('C-section') before?	39	26.9%	75	51.7%	114	78.6%
39. How long will I need to wait for a ‘booking appointment’?	77	54.6%	25	17.7%	102	72.3%
16. Does my private health insurance cover births in this facility?	30	20.5%	75	51.4%	105	71.9%
21. Is shared care offered?	63	48.1%	31	23.7%	94	71.8%
26. Does this facility have a midwifery group practice model?	44	33.8%	48	36.9%	92	70.8%
3. Is there ample parking?	76	52.1%	21	14.4%	97	66.4%
37. How long will I need to wait in the waiting room when I have appointments?	66	45.2%	29	19.9%	95	65.1%
33. How many women have caesarean sections (‘C-sections’) here?	54	37.0%	38	26.0%	92	63.0%
15. If the facility is a public facility, can I be cared for as a private patient?	52	35.9%	36	24.8%	88	60.7%
32. Does this facility supply things like nappies and soap, or will I have to bring them myself?	63	43.2%	25	17.1%	88	60.3%
1. What are the visiting hours?	70	47.9%	17	11.6%	87	59.6%
40. Can I be cared for by a female care provider if I want to?	62	42.5%	24	16.4%	86	58.9%
34. How many women have their labour induced here?	50	34.2%	35	24.0%	85	58.2%
14. Can women from anywhere birth in this facility?	53	36.6%	30	20.7%	83	57.2%
35. How many women have an episiotomy here?	38	26.4%	40	27.8%	78	54.2%
11. I live far away from a maternity facility - is there somewhere nearby where my family can stay?	57	39.9%	20	14.0%	77	53.8%
8. What is the food like?	61	41.8%	14	9.6%	75	51.4%
36. How many women have a vaginal tear here?	42	28.8%	33	22.6%	75	51.4%
5. How many women have a baby here each year?	60	42.0%	9	6.3%	69	48.3%
29. Are childcare facilities available for my other children while I have my baby?	46	31.5%	14	9.6%	60	41.1%
22. Can my baby stay in the nursery on the first night after birth?	40	27.8%	16	11.1%	56	38.9%
23. Are there social areas set-up where I can meet and talk to other mums?	46	31.7%	8	5.5%	54	37.2%

Information items related to policies about the presence of support people in the birth room and about the extent which one’s support people would be made to feel welcome were regarded as important by the overwhelming majority of participants (100 % and 93 % of participants, respectively). Whether other women would recommend a facility to friends was also valued by most participants, with 97 % of participants regarding this as important or extremely important to decision-making.

Approximately 96 % of participants desired information about whether the facility is supportive of a woman choosing her preferred position during labour and birth. Information about the aesthetic quality of the facility (i.e., how ‘nice’ a facility is), and about access to an on-site NICU, were also regarded as important to decision-making about birth facility by more than 90 % of participants.

Information items related to the frequency with which facilities perform certain medical procedures were less universally valued, but were still regarded as important by many participants. For example, 58 % of participants regarded information about a facility’s labour induction rate as important or extremely important to decision-making. Slightly over half of the participants valued knowing the proportion of women who have an episiotomy and who experience a perineal tear during birth (54 % and 51 %, respectively). As many as 63 % of participants valued knowing a facility’s caesarean section rate, and 79 % said that information about access to vaginal birth after caesarean section (VBAC) was important or extremely important to decision-making about birth facility.

Overall, there were few information items that did not hold importance to at least half of the sample. Information about the availability of childcare facilities for one’s other children (41 % agreement) and information about whether one’s baby could sleep in a nursery on the first night after birth (39 % agreement) were among the items considered important by the fewest participants. Information about the availability of designated social areas to interact with other mothers was the least widely valued information item, but even this item was considered important by over one third of participants.

### Other important information items

In all, 77 participants (52.7 %) provided at least one open-ended response when invited to specify further items of key personal importance to decision-making about birth facility. The number of responses provided by each participant ranged from 1 to 10, and 238 responses were provided in total. Some women used their open-ended responses to repeat or elaborate further on domains already covered by the survey of information needs, while others highlighted entirely novel information needs in their open-ended responses. The frequency and proportion of participants providing at least one response in each category, as well as illustrative examples of responses in each category, are provided in Table 3.

**Table 3:** Proportion of participants who provided a response in each additional category (n=146)

**Category**	**Example Responses**	**Freq**	**(%)**
Postnatal care and support	Does the hospital provide support for new mothers once at home?	18	(12.3%)
	What kind of information is provided (i.e. contact details) if we run into trouble once we are at home (i.e. breastfeeding issues)?		
	Staff knowledge of community support services for mothers once they leave hospital?		
Policies and practices related to medical intervention	What is the facility’s C-section rate?	17	(11.6%)
	Does this hospital allow vaginal delivery for breech births?		
	Are there any time periods of labour after which I will need to have intervention?		
Access to water and water immersion	Is it possible to labour in a tub or shower?	15	(10.3%)
	Is there a bath available in the birthing room for during labour?		
	Do you have the facilities, and trained staff, to allow women to have a water birth?		
Infant feeding policies and practices	Is there a IBCLC on staff - can I access them during my stay?	14	(9.6%)
	Will this hospital support mothers if they choose not to breastfeed?		
	Is this hospital BFHI accredited?		
Continuity of care or carer	If I have a long labour, will the same midwife stay with me to the end?	14	(9.6%)
	Will I see the same doctor for every visit?		
	Can I access my midwife team in the few days following birth?		
Choices regarding care providers	What are the guidelines for obstetrician attending birth vs midwife only?	14	(9.6%)
	Does my preferred private obstetrician practise here?		
	Are the staff open to and familiar with alternative methods of birth support? ie. independent midwives, doulas?		
Length of stay and discharge processes	Can I leave as soon as possible after giving birth if I feel confident enough and medically cleared to do so?	12	(8.2%)
	Can I choose to stay in hospital longer if I feel I am not ready to go home?		
	What do we need to do before we can leave (e.g. hearing test, Hep B vaccination, wash baby etc)		
Birth environment and facilities	What labour aids are available from the hospital e.g. fitness balls, aromatherapy burners, CD player, visualisation tools, etc?	11	(7.5%)
	Can I have the lights dimmed for a vaginal birth and my own music playing?		
	Will I have to share a bathroom with another person?		
Policies and practices related to the postnatal hospital stay	Can I keep the baby in bed with me?	10	(6.8%)
	Can I choose to put my baby into the nursery if I feel I need it?		
	Is supervision available for baby while showering/toilet if bathroom is separate to beds?		
Antenatal information and support	Can my midwife visit me at home for any appointments?	10	(6.8%)
	Ability to contact the hospital before the birth if there are any questions or concerns?		
	If ante-natal class is unavailable, where else can I go?		
Facilities and processes for emergency situations	What emergency facilities are available, e.g. operating theatres for if emergency caesarean is necessary	9	(6.2%)
	If my baby needs a transfer, can I go with them?		
	Are staff experienced in looking after high risk women or does the facility cater mainly to low risk women		
Quality of interpersonal care and respect for individual preferences	Will the hospital staff be understanding of my religious beliefs in regards to childbirth?	9	(6.2%)
	Are staff culturally trained?		
	If nursing staff are compassionate, caring and professional?		
Available methods of pain relief	Do you have items available to help with pain e.g. beanbag, ball, shower, bath, Tens machine?	8	(5.5%)
	Do you do 'walking' epidurals?		
	Are the staff open to and familiar with alternative methods of pain management?		
Policies and processes about people in the birth room	Can I have my children present at the birth?	7	(4.8%)
	Is there a limit to how many support people I can have with me during the birth?		
	Can I limit the number of staff present in my birth room?		
Access to psychosocial support	Grief/crisis counselling for you and partner?	5	(3.4%)
	Support services for mothers, e.g. post natal depression, stillbirth?		
	What kind of support does the hospital provide if the mother feels as though they are not coping?		
Costs	What fees/costs might we expect (approximately) for different aspects of care?	5	(3.4%)
	Out of pocket costs - even for public – e.g. scans, antenatal classes etc, car parking, TV connection?		
	How much is the cost of parking? is there weekly parking available?		
Other	Are there vegetarian/vegan food options?	22	(15.1%)
	Is there an exclusion policy? (health/previous births?)		
	Is a gown/clothing provided to give birth in or do I need to bring my own?		

Information items pertaining to postnatal care and support were most frequently cited. Participants typically wished to know how much in-home care is provided by the facility following discharge and what information resources and contacts are provided for use once at home. Information about policies and practices related to medical intervention were also frequently mentioned by participants, as was information about whether women could access water and water immersion during labour and/or birth.

### Discussion and conclusions

The objective of this study was to inform the development of a decision support tool for birth facility in Queensland, Australia by identifying the information needs of potential users. One hundred and forty-six women residing in Queensland who were pregnant and/or had recently had a baby were surveyed about their information needs when choosing a birth facility. This study found that the women surveyed attached a high level of importance to many different pieces of information for decision-making about birth facility. On average, women identified 30 out of 42 information items as important or extremely important, and many suggested further information items not covered in the survey. These findings suggest that previous studies which have examined the attributes that consumers value in a birth facility using only limited lists of criteria e.g., [11-13] may have neglected many attributes that are important to this population.

The study findings also highlight trends pertaining to the most and least universally desired information for decision-making about birth facility. The most widely valued information items related to policies about support people, other women’s recommendations about the facility, freedom to choose one’s preferred position during labour and birth, the aesthetic quality of the facility, access to on-site neonatal intensive care, postnatal care and support, policies related to medical intervention, and access to water immersion. Information items related to the actual frequency with which facilities perform certain medical procedures (e.g., induction of labour, episiotomy, caesarean section) were less universally valued, as were information items related to the frequency with which certain outcomes (e.g., perineal tearing) are experienced by women birthing in facilities.

Notably, these patterns in women’s responses may be strongly influenced by their assumptions about the extent to which rates of certain procedures or outcomes are determined by non-clinical factors. For example, someone who assumes that the likelihood of a certain procedure being performed depends only on the clinical characteristics of the woman receiving care may attach less importance to knowing a facility’s rate of performing that procedure than someone who assumes that institutional, clinician or other factors also determine the likelihood of that procedure being performed. Further examining this potential association is a worthwhile avenue for future research and would provide insight into how necessary it is to implement awareness-raising strategies about clinical practice variation simultaneously with tools to support decision-making about birth facility.

A notable limitation of this study is that, while considerable efforts were made to maximise the diversity of the sample through multiple channels of recruitment and multiple methods of study participation, a convenience sampling framework was adopted in recruiting participants. It is likely that the resulting sample under-represented some sub-populations of women, including younger women, women who identify as Aboriginal or Torres Strait Islander, women with lower levels of formal education and women who speak a language other than English at home, although data on the socio-demographic characteristics of the target population (i.e., women who are pregnant or who have had a baby in the last two years) are not available to test this. While the extent of the impact of this limitation on the reliability and validity of the current findings is unclear, replicating this study using a random sampling framework with over-sampling of groups typically under-represented in survey-based research is desirable.

In this study, there was also insufficient equality in distribution of participants across key socio-demographic categories (e.g., parity, age, education level) to allow for the examination of intergroup differences in information needs. It would be reasonable to assume that women vary in their preferences for information, and further research examining the priorities of different sub-populations would provide data to inform any necessary tailoring of decision support tools. However, again, the extremely high endorsement of many of the items provided to participants suggests a degree of universality in information needs, at least within this sample of pregnant and postnatal women.

Notwithstanding these limitations, there are clear, immediate implications of our findings. The findings are useful for directly informing the development of decision support tools about birth facility. Specifically, the relative importance of different information items to this group of women can be used to prioritise a list of indicators on which to provide specific facility information. Ensuring that the content of decision support tools reflects the needs of end users in this way is a critical strategy to ensuring their relevance, uptake and utility [10]. Developing strategies for ensuring continued responsiveness to the information needs of the users of such tools (e.g., allowing users to a request that specific information be added) is also important and would maximise the utility of the tool over time or among populations whose information needs may not have been adequately captured in the current study.

While knowing and responding to the information priorities of users of decision support tools is essential, the present finding that pregnant and postnatal women attach high value to many different information items also introduces challenges for the developers of decision support tools. Content-heavy decision support tools can cause information overload among users, even if the information is desired, which can both be disengaging and undermine utility [21]. Accordingly, tools would benefit from tailoring mechanisms which allow users to limit information to that which is personally relevant. Interactive, computer-based decision support tools lend themselves particularly well to this sort of information customisation [22], and the present findings have informed the development of one such decision support tool in Queensland, Australia [23].

These findings also have applications for providers who are involved in counselling pregnant women and families about their options for birth facility and/or providing referrals to birth facilities. Specifically, providers can use these findings to guide information sharing and communication as part of shared decision-making about birth facility, while ensuring they remain sensitive to, and responsive to, individual differences in information needs.

## Endnotes

^a^In this instance, a standard primipara is defined as “a woman, 20–34 years of age, who has given birth for the first time, free of obstetric and specific medical complications and pregnant with a singleton pregnancy at term (37 weeks 1 day–40 weeks 6 days gestation), with a non-small for gestational age (greater than tenth percentile) infant and a head first (cephalic) presentation” [6].

## Abbreviations

BFHI, Baby Friendly Hospital Initiative; GP, General Practitioner; IBCLC, International Board Certified Lactation Consultant; NICU, Neonatal Intensive Care Unit; SCN, Special Care Nursery; SD, Standard Deviation; VBAC, Vaginal Birth After Caesarean.

## Competing interests

Neither Rachel Thompson nor Aleena Wojcieszek has any financial or personal relationships with other people or organisations that could inappropriately influence this work.

## Authors’ contributions

RT conceived the study, contributed to its design and to data acquisition, performed quantitative data analysis, contributed to qualitative data analysis, and drafted the manuscript. AW contributed to the study design and coordination, contributed to data acquisition, contributed to qualitative data analysis and contributed to revisions of the manuscript. Both authors read and approved the final manuscript.

## Pre-publication history

The pre-publication history for this paper can be accessed here:

http://www.biomedcentral.com/1471-2393/12/51/prepub
